# The influence of sense of agency on learning structured regularities: an artificial grammar learning study

**DOI:** 10.1007/s00426-026-02308-3

**Published:** 2026-05-28

**Authors:** Romina Haxhi, Mateusz Woźniak, Agnieszka Wykowska

**Affiliations:** 1 Social Cognition in Human-Robot Interaction Unit, Italian Institute of Technology, Genoa, Italy; 2 University of Ferrara, Ferrara, Italy; 3 Institute of Applied Psychology, Jagiellonian University, Kraków, Poland; 4 Institute for Advanced Study, Technical University of Munich, Lichtenbergstrasse 2 a, D-85748 Garching, Germany

## Abstract

In many real-life situations learning is an active process of engaging with new information, experimenting and integrating new experiences to build meaningful understanding. However, learning can have either a more active or more passive form and several factors may influence how effectively individuals learn new information in both cases. One such factor is the sense of agency, which refers to the subjective feeling of control over one’s actions, and it distinguishes active experience from passive observation. The present study aims to investigate whether differences in the sense of agency affect learning, as evaluated using the artificial grammar learning task. Participants were assigned to either an active condition, where they had full control over responses, or a passive condition, where they mostly passively observed the computer performing the task and were learning through observation. At the end of the experiment, they rated their perceived control. Results showed that participants in the active condition reported a stronger sense of agency and demonstrated higher accuracy from the very beginning of the task compared to those in the passive condition. Although both groups improved with repeated trials at a similar rate, the initial advantage for the active group persisted throughout the experiment. Overall, our study suggests that sense of agency can influence learning, and that learning is more effective when individuals feel in control of their actions.

## Introduction

The sense of agency (SoA) is described as the individual’s subjective feeling of control over their actions and the perception that these actions can influence outcomes in the environment (Haggard & Chambon, 2012; van der Wel et al., 2012; Wen & Imamizu, 2022). In contrast to a similar concept of locus of control, which refers to a relatively stable belief about whether outcomes in life are determined by one’s own actions or by external forces (Rotter, 1966), sense of agency reflects a situational and instantaneous experience of initiating actions and causing their immediate effects. Distinguishing these constructs is particularly important when examining learning in controlled experimental settings, where situational experiences of agency may vary independently of broader control beliefs. Experiencing a strong SoA has been shown to enhance motivation, attention, and cognitive engagement (Penton et al., 2018; Van den Bussche et al., 2020), whereas a diminished SoA is associated with lower attentional involvement, impaired information processing, and weaker memory consolidation (Houser et al., 2022; Wen et al., 2016). These findings suggest that SoA is not just a subjective feeling but an important process that shapes how individuals perceive and interact with their environment. It depends on predictive and feedback-based processes that allow individuals to connect actions with their consequences, detect discrepancies between expected and actual outcomes, and adjust behaviour accordingly (Haggard & Chambon, 2012; Synofzik et al., 2013). From this perspective, experiencing agency could serve as a bridge between cognitive engagement and the active construction of knowledge.

Learning is recognized more as an active and constructive process rather than a passive absorption of information (Bada & Olusegun, 2015). According to constructivist learning theorists such as Bruner and Piaget, knowledge deepens when individuals integrate new information with existing knowledge (Bruner, 1966; Anthony, 1996; Pakpahan & Saragih, 2022). This view emphasizes that learning could benefit from the opportunity to explore and continuously reorganize prior knowledge (Chand, 2024). In parallel with this view, the self-determination theory emphasizes the importance of autonomy and perceived control in developing deeper and more sustainable learning (Legault, 2017; Ryan & Deci, 2017). These perspectives together suggest that learning is a process determined not only by the information provided but also by the degree of participation and control individuals experience throughout this process. Consequently, agency may be important for learning, as it could transform experience from passive observation into active understanding. (Dunlop, 2003; Schoon and Heckhausen 2019).

Recently, SoA has been investigated as a factor shaping how learners engage with and extract structured patterns from their environment. New research is showing that experiencing a strong SoA can bias attentional allocation toward intended outcomes and enhance subsequent action regulation, suggesting that perceived control shaped how individuals selectively engage with and process information in structured environments (Nakashima, 2019; Ren et al., 2023). Moreover, when learners perceive autonomy over their actions, they are more likely to engage in active exploration such as testing alternatives and hypotheses, experimenting with strategies, and navigating multiple solution paths (Liu et al., 2025). Furthermore, exploration is particularly important in learning contexts that require pattern recognition, problem solving and adaptation, as it allows learners to generate hypotheses, evaluate feedback and refine their understanding (Joshi et al., 2024).

One useful paradigm to investigate how people detect structure in perceived stimuli is Artificial Grammar Learning (AGL) (Trotter et al., 2020). In a classic AGL task, participants are exposed to sequences of letters or symbols generated from a finite-state grammar (Reber, 1967). Subsequent studies have also used other types of stimuli such as non-linguistic (shapes and colours) (Conway & Christiansen, 2006; Soares et al., 2021), auditory (tones) (Emberson et al., 2006; Heimbauer et al., 2018), or time based (durations) (Prince et al., 2018), each constructed according to specific underlying rules that are not revealed to the participant during the exposure phase (Reber, 1967). Next, the participants complete a test phase in which they are informed that all sequences have a certain “nature” and they have to identify whether the new sequences match the previous sequence based on their “gut feeling” or intuition. (Gillis et al., 2022; Westphal-Fitch et al., 2018). Performance above chance level suggests that participants have learned at least some of the underlying features or regularities (Guillemin & Tillmann, 2021).

AGL provides a highly controlled and reproducible framework in which the structure and complexity of grammatical and ungrammatical strings of symbols can be systematically varied to test specific hypotheses about learning mechanisms (Reber, 1967; Westphal-Fitch et al., 2018). This flexibility makes this paradigm particularly well suited for examining how factors such as control, exploration and action-outcome contingencies influence the acquisition of structured regularities as it provides a setting in which learners can form and evaluate hypotheses about underlying patterns. Research on the informational advantages of learning by selection suggests that when learners can choose which information to sample, they tend to gather more informative data and focus on observation that is most useful for refining their hypothesis (Markant & Gureckis, 2014). Sequential hypothesis-testing theory provides a formal account of this process, proposing that learners generate provisional hypotheses and iteratively update them based on observed outcomes. Learning proceeds trial by trial, with hypotheses guiding what evidence is sampled and new observations used to confirm, refine or reject current beliefs (Markant & Gureckis, 2014).Because AGL involves a hidden rule structure that affords multiple interpretations of sequence regularities, it offers a good framework for examining these hypothesis-testing dynamics (Reber, 1967). Nonetheless, despite research using AGL to study learning, the role of agency in shaping structured learning outcomes remains underexplored. Recent theories suggest that autonomy and perceived control enhance learning by aligning internal predictions with feedback from the environment, thereby reinforcing motivation and cognitive engagement (Dutta, 2025). From this perspective, SoA is not merely a subjective feeling of control but an active component that shapes how information is processed and internalized (Stern et al., 2022).

The present study aimed to investigate how control and the resulting sense of agency influence learning through an AGL task by comparing active and passive learning conditions. We hypothesized that participants who are part of the group with full control over the choices, and consequently a higher sense of agency, will be better at identifying the grammar rules compared to the participants who are part of the group with a limited sense of agency. Furthermore, we expected that participants in the active condition would report a significantly higher sense of agency, confirming that the experimental manipulation successfully altered perceived control.

## Methods

### Participants

In total, there were one hundred and seventy-four participants, eighty-seven individuals per condition (active learning group vs. passive learning group), recruited from Prolific, an online participant recruitment platform. Their age varied from 18 to 30 years (*M* = 24.8, *SD* = 3.12). The group included 88 females, 86 males, and 2 individuals who declined to specify their gender. Participants were recruited via Prolific and compensated at an average rate consistent with Prolific’s recommended pay (hourly rate of 8.83). The active condition lasted approximately 30 min on average, while the passive condition lasted approximately 25 min. The study included five attention – check questions that appeared at random times. Participants would be excluded if they had failed more than two attention checks. No participants failed more than one attention check, so no exclusions were made.

The criteria to be a part of this experiment were for the participants to be within the 18 to 30 years old age range and speak English fluently. To ensure that no participant was exposed to both conditions, an exclusion criterion was applied for the passive condition, where individuals who had already participated in the experiment with the active condition were not eligible to participate. This exclusion criterion was intended to prevent any participant from experiencing both conditions, which could have introduced carry-over effects. The order of the active versus passive conditions was not fixed. The total sample was divided into three batches of 30 participants each. The order of conditions for these batches was as follows: active-passive, passive-active and active-passive.

The study was approved by the local Ethical Committee (Comitato Etico Regione Liguria) and was conducted in accordance with the Code of Ethics of the World Medical Association (Declaration of Helsinki). Each participant provided informed consent before taking part in the experiment by clicking on the “Yes” option at the beginning of the experiment.

### Design

The experiment employed a 2 (group: active and passive, between-subject factor) x 8 (blocks: 1–8, within-subject factor) mixed factorial design. The main dependent variable was the average performance measured as the percentage of correct responses. The block factor was included to track changes in participants’ accuracy over the course of the experiment. There were a total of 164 trials which were divided into 8 blocks of 20 trials, except for the final block, which consisted of 24 trials. This was done to check end-of-task level of performance, allowing for a more accurate estimate of participants’ final accuracy and learning outcome of the artificial grammar.

All participants completed the AGL task with one grammar throughout the experiment and therefore there was no change in grammar rules across blocks.

### Procedure

The participants were first shown written instructions describing their task and the goal of the experiment, which was to learn the hidden set of grammatical rules, allowing them to identify the “grammatical” string of symbols. The task was to select one of the strings (presented laterally at the top of the screen) by moving the arrow towards it. They could move the arrow by pressing the left or right keys on the keyboard at least ten times to move the arrow towards the string they thought was the correct one. A single key press moved the arrow diagonally up-and-to-the-left or up-and-to-the-right (depending on the pressed key). If the arrow reached the screen height of the strings, but was between them, then subsequent keypresses moved the arrow left or right. The trial ended when the arrow reached one of the strings. Participants were not able to move the arrow to areas of the screen not related to the task. Once they made their choice, they received written feedback if it was the correct string or not, which would remain on screen for 1000ms. In the active condition, the participants had full control over the arrow during all of the trials.

On the contrary, in the passive condition, at the beginning of the experiment, they were instructed that in some of the trials they would be able to control the arrow and in others the computer would do so. After each 8 computer-controlled trials, participants completed 2 active trials. In the computer-controlled trials the arrow moved towards one of the strings by itself, without participants pressing any key. The arrow moved in steps, similarly to a situation in which a participant would be pressing keys on a keyboard. The duration between each movement was constant and was calculated based on average time of participants’ responses from a pilot study. After the computer made the selection, feedback was presented similarly as in the active condition. In the trials that participants had full control, they moved the arrow towards one of the strings in the same way as in the active condition.

**Fig. 1:**
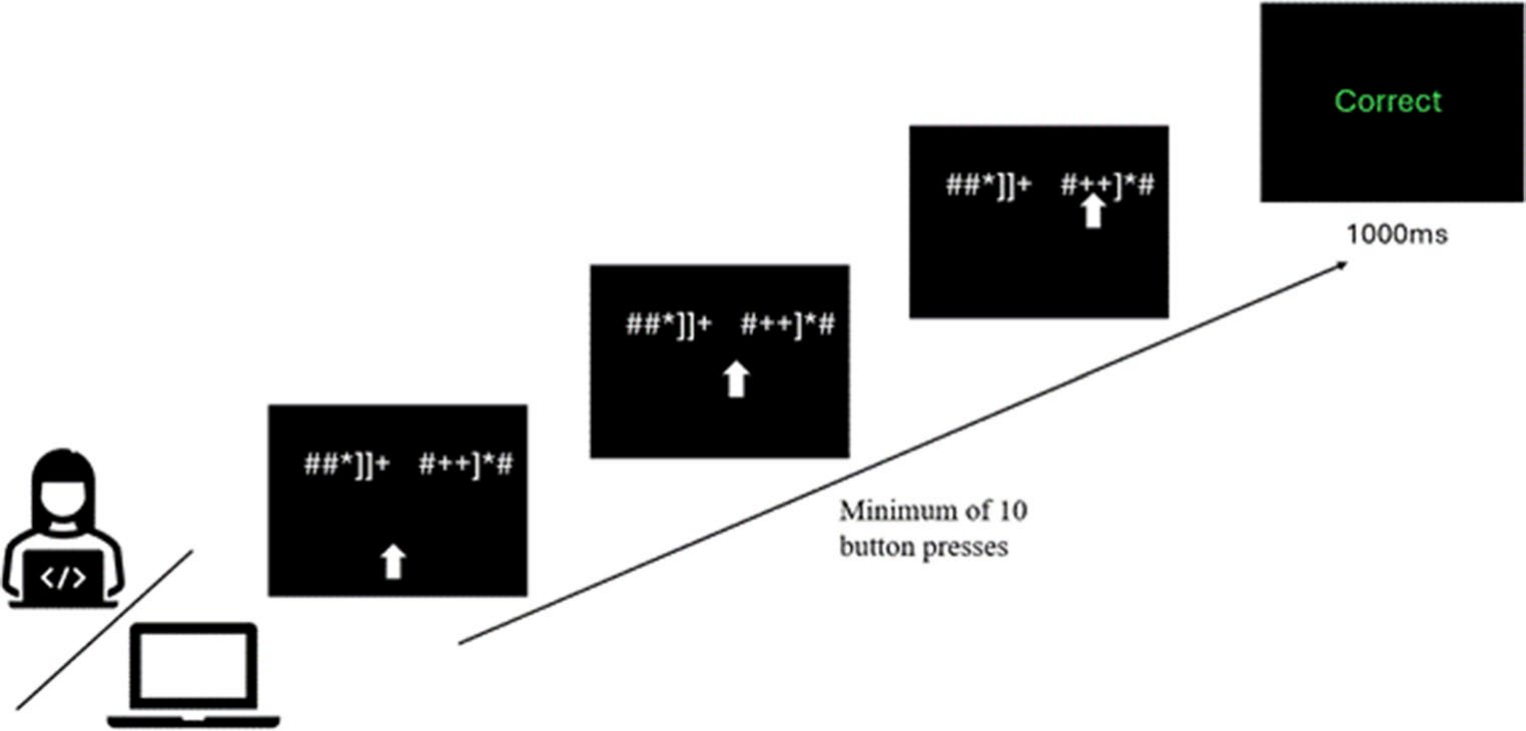
A representation of the trial sequence

In the passive condition, the computer’s arrow movements were programmed to gradually improve over the course of the session. At the start, responses were at chance level, and accuracy increased linearly across trials so that by the end of the experiment performance reached approximately 90.5%. This was implemented in PsychoPy by assigning a trial-by-trial probability of responding correctly. On each trial, the computer used this probability to determine whether to select the correct or incorrect side, ensuring that participants observed progressively more accurate choices and could track the apparent improvement over time (Fig. 1).

Upon completing the experiment, participants were required to rate their sense of agency over the arrow’s movement. This was measured using a visual analogue scale (VAS) ranging from 1 = “I had no control at all” to 100 = “I was fully in control”. They were also asked to provide a brief open-ended explanation of their rating. Finally, to assess awareness of the grammar, they were asked “What do you think were the rules that the correct words followed?”

### Materials and apparatus

**Fig. 2:**
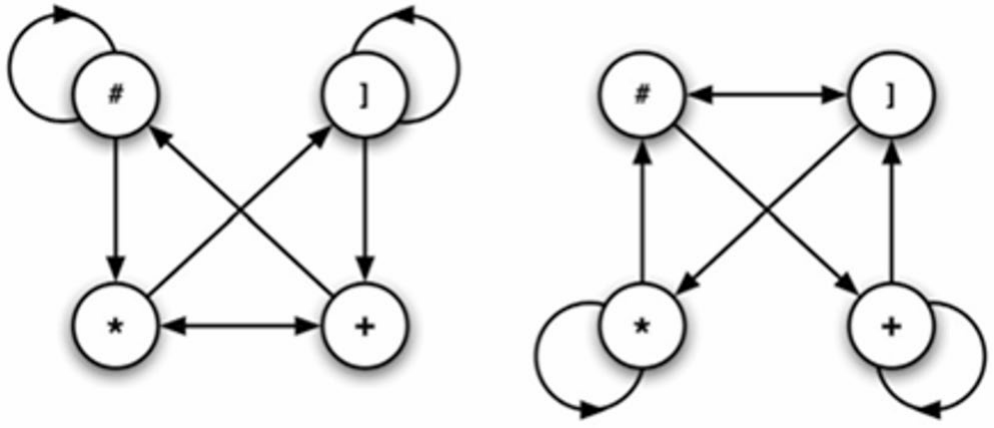
The two finite-state grammars used to generate the sequences for Grammar A and Grammar B. Adapted from “Intuitive decision making in complex situations: Somatic markers in an artificial grammar learning task,” by D. J. Bierman, A. Destrebecqz, and A. Cleeremans, Cognitive, Affective, & Behavioral Neuroscience, 5(3), 297–305 (2005)

The experiment was programmed in PsychoPy v.2024.2.5 using graphical user interface with fragments of code written in Python. Afterwards it was translated into JavaScript using PsychoJS and hosted on the Pavlovia hosting service. Participants completed the task on their own computers. The stimuli used in this experiment consisted of one correct and one incorrect string of symbols consisting of the following symbols: #, ], * and +. Both strings had 6 symbols in total and were generated based on two finite state grammars illustrated in Fig. 2. All pairs included one string generated from Grammar A, from which the correct words were calculated, and one string generated from Grammar B, from which the incorrect words were generated. The side on which the correct word would appear was randomized. The colour of the background was set to black, and the colour of the arrow and stimuli was white. The feedback colour was green for correct and red for incorrect. The size of the symbols was 0.05 PsychoPy norm units, and the height and width of the arrow were both 0.1 PsychoPy norm units. The creation of these strings followed the rules that Bierman et al. (2005) had in their original experiment. To generate the string of symbols, the first component was selected at random. The following elements were chosen by randomly selecting a path from the current node, following the transition probabilities set by the grammar map until all six elements had been generated. Moreover, the occurrence of strings with more than two consecutive identical symbols after the first self-transition was set to zero.

## Results

### Learning across blocks and conditions

**Fig. 3:**
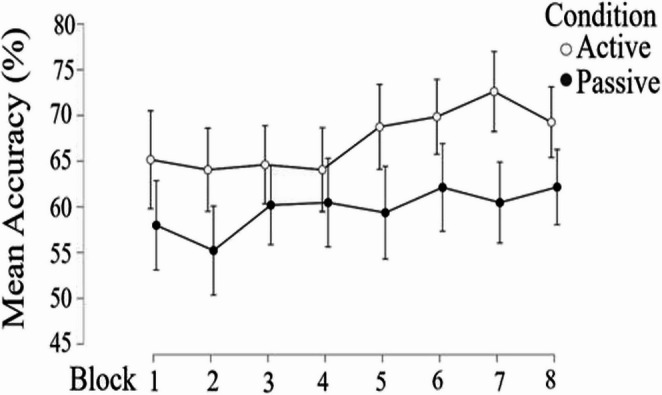
Mean accuracy (%) across eight learning blocks for the active and passive conditions

A 2 (Condition: active vs. passive) × 8 (Block 1–8) mixed ANOVA was conducted on accuracy to examine changes in learning performance over time. The analysis revealed a significant main effect of Condition, *F*(1, 172) = 11.10, *p* = .001, η² = 0.022, with overall accuracy (higher for active) differing between active and passive groups. There was also a significant main effect of Block, *F*(7, 1204) = 2.26, *p* = .027, η² = 0.008, indicating that performance varied across blocks. The Block × Condition interaction was not significant, *F*(7, 1204) = 0.70, *p* = .672, suggesting that the pattern of change across blocks was similar for both groups (Fig. 3).

### Sense of agency ratings

**Fig. 4:**
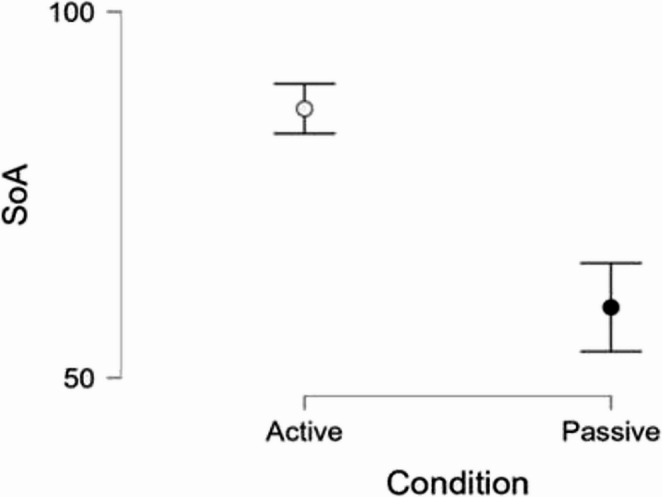
The difference in the reported sense of agency (SoA) between the active and passive condition groups

To examine the subjective experience of control, an independent sample *t*-test was performed on SoA ratings. Participants in the active condition reported significantly higher SoA than those in the passive condition, *t*(170) = 7.78, *p* < .001 (Fig. 4).

## Discussion

The present study explored how agency impacts learning by using an AGL task by comparing active and passive learning conditions. Consistent with our hypothesis, we found that participants in the active condition, who had full control over their responses, demonstrated significantly higher accuracy in categorizing strings of symbols according to a rule, compared to participants in the passive condition. Additionally, participants in the active group also reported a stronger subjective sense of agency, confirming the effectiveness of the experimental manipulation. Most importantly, we observed this boost in performance immediately at the beginning of the experiment. Accuracy in the active condition was already higher in Block 1 and remained consistently higher throughout all eight blocks. Although both groups improved over time, the lack of a significant interaction between them suggests that the rate of improvement across blocks was similar between groups. Therefore, the key difference is in the initial stage of learning suggesting that having full control from the outset appears to provide an early advantage that participants maintain as task progresses. This could also suggest that SoA has a continuous and consistent beneficial effect on learning. Such a pattern aligns with the idea that agency can support early information acquisition or engagement mechanisms, which may then stabilize as task demands become more familiar.

A combination of several cognitive mechanisms related to rule discovery and hypothesis testing could have contributed to the enhanced performance observed in the active condition. When learners can choose which strings to sample, they can directly test predictions derived from their current hypotheses, immediately reinforcing or revising them based on observed outcomes. This sequential, hypothesis-guided approach allows predictions to be directly and actively compared with outcomes, reinforcing correct rules, and prompting revision of incorrect ones (Markant & Gureckis, 2014). Furthermore, selecting and responding to strings could engage distinctive sensorimotor associations between actions and outcomes, which can help structure learning by organizing information around self-generated action- consequence relationships and clarifying causal links between choices and feedback, thereby making feedback more informative (Markant et al., 2016). At the same time, agency supports goal-directed exploration within the rule space, directing attention toward rule-relevant features and fostering the integration of relational information among symbols (Markant et al., 2016).

Furthermore, selecting and responding to strings may facilitate learning not simply by strengthening action–outcome associations, but by enabling more efficient hypothesis testing. In the active condition, participants can strategically select strings that are informative with respect to their current hypothesis. For example, once a learner has ruled out a negative hypothesis (e.g., that the sequence “++#” in incorrect), they can deliberately avoid strings containing “++#” and instead test alternatives that help refine a positive hypothesis about the correct rule. In contrast, in the Passive condition, since the string selection is done by the computer, it may continue to present strings containing “++#”, even after the learner has already rejected the hypothesis. These trials are therefore less informative as they do not allow the learner to actively test emerging positive hypotheses. As a result, active control over sampling may accelerate by aligning evidence acquisition with the learner’s current hypotheses.

In addition, the results of our study are consistent with constructivist theories of learning (Bruner, 1966; Anthony, 1996) and self-determination theory, which emphasizes the role of autonomy and perceived control in deepening cognitive engagement (Legault, 2017; Ryan & Deci, 2017). The higher accuracy observed in the active group suggests that participants who can actively make choices are more effectively able to internalize underlying structural patterns, confirming the hypothesized role of agency in facilitating learning.

On a more practical level, these results could be potentially useful for educators and designers in structuring learning experiences that strategically combine learner’s SoA and also instructional guidance. Our findings suggest that increasing the SoA of the learners can lead to an immediate advantage in learning performance. This could suggest that in the early stages of instruction such as when learners are being introduced to new concepts, unfamiliar material or new tools, providing opportunities to make choices and exercise some control over their interaction with the content could further support learning outcomes. This interpretation is consistent with findings from Ren and Gentsch (2023), who showed that a heightened SoA is linked to a greater action readiness and more proactive responding, suggesting that learners who perceive greater control over outcomes may engage more actively with instructional materials. Similarly, a study manipulating levels of student agency in a game-based learning environment found that students in a high-agency conditions, who could freely navigate and explore the learning environment, demonstrated greater engagement and exploration behaviours. In contrast, students in low-agency conditions achieved higher learning gains but exhibited more unproductive behaviours (Sawyer et al., 2017). Therefore, a right balance between boosting SoA and guidance to eventually focus on relevant information rather than distraction needs to be sought. This aligns with the view that agency is integral to learners’ ability to regulate control and monitor their own learning, supporting self-directed goal setting and strategy use (Code, 2020). This could be used for educational software, classroom instruction and emerging learning tools, suggesting they should offer exploratory, learner-controlled modes early on and transition toward more scaffolded, feedback-driven support over time.

### Limitations and future directions

While the findings provide evidence for the beneficial role of agency, several limitations should be taken into consideration. First, all participants were recruited and tested online which could have led to differences in how focused and engaged they were during the experiment. It is possible that the subjects paid more attention at the beginning of the task than later on. Whereas in a controlled laboratory setting, where distractions are minimized and the task is the primary focus, sustained engagement could be higher. Second, the age of the participants was limited to 18 to 30 years. This age range was chosen to ensure a relatively consistent level of cognitive functioning and familiarity with technology across participants, lowering the variability linked to developmental or age-related differences.

Another limitation could be the lack of counterbalancing the grammars between the conditions. In the present design, Grammar “A” served always as the target rule set for all participants meaning it was always the correct grammar and grammar “B” was always the incorrect one. Although counterbalancing grammar assignment across groups could have removed any potential grammar-specific bias, both grammars were presented equally and in randomized order within both conditions, and the correct-side presentation was also randomized, ensuring that any potential differences in grammar difficulty were distributed across groups. Therefore, even if counterbalancing was not implemented, it is unlikely that this design feature systematically influenced the group differences observed in learning performance. This choice was directly based on Bierman et al. (2005), from whom the AGL task and grammar structures were adapted.

Future studies could address these limitations by examining the effect in the lab (rather than online) and across a wider range of age. This paradigm and its variations could also be explored with different populations, such as children, older adults, or people with learning difficulties, in order to learn more about how age and other individual characteristics affect the link between agency and learning. Another option to explore would be to include a fully passive condition as well. In the condition considered passive in this experiment, the participants still had around 20% control over the arrow and they reported their SoA at around 50 points, therefore a fully passive condition might have a different effect on their performance. Further, it would be interesting to explore the interaction between agency and other factors that influence learning, for example, attentional load or task difficulty. Finally, it would be of interest to examine if SoA also affects other cognitive mechanisms, such as memory.

## Conclusions

In conclusion, this study explored how agency influences learning, using an artificial grammar learning task. The study aimed to clarify how feelings of autonomy and control support the unconscious acquisition of structured knowledge by examining performance under two different levels of agency. The results showed that participants who had full control over their actions performed more accurately than those with limited control. This finding supports the idea that when learners experience a stronger sense of agency, they might become more attentive and motivated, which enhances their ability to learn. Although both groups improved over time, the consistent advantage of the active group suggests that agency strengthens the quality of learning since the beginning rather than its rate of development. These findings emphasize the importance of learner involvement in experimental design. They also show how the subjective experience of control influences cognitive performance and how a higher SoA may provide a cognitive advantage in learning.

## Public significance statement

The feeling of being in control over one’s actions can shape how quickly individuals acquire new information. This study shows that when people experience a stronger sense of agency, they show more effective learning from the very beginning of the task and maintaining this advantage as the task progresses. These findings show that strengthening a learner’s sense of agency may enhance learning outcomes in possible educational and everyday contexts.

## Data Availability

Data will be available on OSF upon acceptance of the paper.
